# Supplementary material for: Assessment tools and incidence of hospital-associated disability in older adults: a rapid systematic review
Source: PeerJ. 2023 Oct 19;11:e16036. doi: 10.7717/peerj.16036 (PMC10590575; doi:10.7717/peerj.16036)
Supplement: Supplemental Information 1 — The research strategy of all databases, detailed table describing the calculated and estimated incidences of HAD categorized per ADL task and set of tasks, the flow diagram and the individual question rating JBI Checklist. [file peerj-11-16036-s001.docx]

ADDITIONAL MATERIAL

Additional material Table 1. Search strategy.

Medline (via Ovid) search.

| **Number** | **Query** | **Results the 26 Aug 2021** |
| --- | --- | --- |
| 1 | Hospitalisation.ti,ab. | 17,353 |
| 2 | exp *Hospitalization/ | 93,562 |
| 3 | hospitalization.ti,ab. | 137,158 |
| 4 | Hospital.ti,ab. | 1,075,670 |
| 5 | Hospitalized.ti,ab. | 112,702 |
| 6 | Hospitalised.ti,ab. | 13,156 |
| 7 | In-Hospital.ti,ab. | 98,939 |
| 8 | Inhospital.ti,ab. | 1,870 |
| 9 | Inpatient.ti,ab. | 88,351 |
| 10 | 1 or 2 or 3 or 4 or 5 or 6 or 7 or 8 or 9 | 1,300,482 |
| 11 | functional.ti,ab. | 1,338,546 |
| 12 | Disability.ti,ab. | 168,863 |
| 13 | disabled.ti,ab. | 24,221 |
| 14 | Activity of daily living.ti,ab. | 2,185 |
| 15 | Activities of daily living.ti,ab. | 28,860 |
| 16 | Activity of daily life.ti,ab. | 482 |
| 17 | Activities of daily life.ti,ab. | 1,403 |
| 18 | exp *"Activities of Daily Living"/ | 45,453 |
| 19 | activities.ti,ab. | 755,046 |
| 20 | daily.ti,ab. | 557,713 |
| 21 | living.ti,ab. | 373,448 |
| 22 | 19 and 20 and 21 | 32,687 |
| 23 | Activity.ti,ab. | 2,802,780 |
| 24 | daily.ti,ab. | 557,713 |
| 25 | living.ti,ab. | 373,448 |
| 26 | 23 and 24 and 25 | 8,758 |
| 27 | Participation.ti,ab. | 159,573 |
| 28 | Activity.ti,ab. | 2,802,780 |
| 29 | limitation.ti,ab. | 90,604 |
| 30 | 28 and 29 | 11,618 |
| 31 | 11 or 12 or 13 or 14 or 15 or 16 or 17 or 18 or 22 or 26 or 27 or 30 | 1,699,597 |
| 32 | prevalence.ti,ab. | 683,571 |
| 33 | frequency.ti,ab. | 878,298 |
| 34 | burden.ti,ab. | 230,392 |
| 35 | exp Random Allocation/ | 105,786 |
| 36 | random*.ti,ab. | 1,247,309 |
| 37 | frequencies.ti,ab. | 197,525 |
| 38 | inciden*.ti,ab. | 951,438 |
| 39 | incidence.ti,ab. | 805,068 |
| 40 | risk.ti,ab. | 2,320,682 |
| 41 | hazard ratio.ti,ab. | 108,340 |
| 42 | hazard ratios.ti,ab. | 29,363 |
| 43 | odds.ti,ab. | 363,083 |
| 44 | chance.ti,ab. | 69,946 |
| 45 | percent.ti,ab. | 295,410 |
| 46 | percentage.ti,ab. | 403,689 |
| 47 | percentages.ti,ab. | 66,053 |
| 48 | rate.ti,ab. | 2,216,830 |
| 49 | rates.ti,ab. | 1,158,092 |
| 50 | occurrence.ti,ab. | 386,376 |
| 51 | responsiveness.ti,ab. | 105,969 |
| 52 | sensitivity to change.ti,ab. | 2,604 |
| 53 | 32 or 33 or 34 or 35 or 36 or 37 or 38 or 39 or 40 or 41 or 42 or 43 or 44 or 45 or 46 or 47 or 48 or 49 or 50 or 51 or 52 | 7,901,067 |
| 54 | older.ti,ab. | 474,685 |
| 55 | elderly.ti,ab. | 259,926 |
| 56 | geriatric.ti,ab. | 44,737 |
| 57 | exp *Aged/ | 26,580 |
| 58 | aged.ti,ab. | 623,417 |
| 59 | frail.ti,ab. | 13,694 |
| 60 | frails.ti,ab. | 13 |
| 61 | exp *Frailty/ | 4,180 |
| 62 | frailty.ti,ab. | 16,886 |
| 63 | frailness.ti,ab. | 20 |
| 64 | ageing.ti,ab. | 45,000 |
| 65 | senior.ti,ab. | 36,394 |
| 66 | elders.ti,ab. | 9,003 |
| 67 | 54 or 55 or 56 or 57 or 58 or 59 or 60 or 61 or 62 or 63 or 64 or 65 or 66 | 1,256,648 |
| 68 | hospital-associated.ti,ab. | 1,276 |
| 69 | hospital associated.ti,ab. | 1,276 |
| 70 | hospital-acquired.ti,ab. | 10,652 |
| 71 | hospital acquired.ti,ab. | 10,652 |
| 72 | hospital-related.ti,ab. | 806 |
| 73 | hospital related.ti,ab. | 806 |
| 74 | hospitalization-associated.ti,ab. | 309 |
| 75 | hospitalization associated.ti,ab. | 309 |
| 76 | hospitalisation-associated.ti,ab. | 38 |
| 77 | hospitalisation associated.ti,ab. | 38 |
| 78 | hospitalization-acquired.ti,ab. | 3 |
| 79 | hospitalization acquired.ti,ab. | 3 |
| 80 | hospitalisation-acquired.ti,ab. | 1 |
| 81 | hospitalisation acquired.ti,ab. | 1 |
| 82 | hospitalization-related.ti,ab. | 220 |
| 83 | hospitalization related.ti,ab. | 220 |
| 84 | hospitalisation-related.ti,ab. | 28 |
| 85 | hospitalisation related.ti,ab. | 28 |
| 86 | before hospitalisation.ti,ab. | 105 |
| 87 | before hospitalization.ti,ab. | 1,090 |
| 88 | after hospitalisation.ti,ab. | 434 |
| 89 | after hospitalization.ti,ab. | 3,890 |
| 90 | (functional deterioration adj3 hospital*).ti,ab. | 20 |
| 91 | (hospital* adj3 functional loss).ti,ab. | 13 |
| 92 | (functional status adj3 Hospital*).ti,ab. | 342 |
| 93 | (los* adj3 independence).ti,ab. | 1,369 |
| 94 | (exposure adj2 hospital*).ti,ab. | 797 |
| 95 | (functional decline adj4 hospital*).ti,ab. | 313 |
| 96 | 68 or 69 or 70 or 71 or 72 or 73 or 74 or 75 or 76 or 77 or 78 or 79 or 80 or 81 or 82 or 83 or 84 or 85 or 86 or 87 or 88 or 89 or 90 or 91 or 92 or 93 or 94 or 95 | 21,232 |
| 97 | 10 and 31 and 53 and 67 and 96 | 740 |

Embase search.

| **Number** | **Query** | **Results the 26 Aug 2021** |
| --- | --- | --- |
| 1 | hospitalisation:ti,ab | 28,806 |
| 2 | 'hospitalization'/exp | 421,223 |
| 3 | hospitalization:ti,ab | 230,090 |
| 4 | hospital:ti,ab | 1,708,899 |
| 5 | hospitalized:ti,ab | 175,158 |
| 6 | hospitalised:ti,ab | 20,314 |
| 7 | 'in hospital':ti,ab | 159,194 |
| 8 | inhospital:ti,ab | 6,247 |
| 9 | inpatient:ti,ab | 151,612 |
| 10 | #1 OR #2 OR #3 OR #4 OR #5 OR #6 OR #7 OR #8 OR #9 | 2,125,565 |
| 11 | functional:ti,ab | 1,651,619 |
| 12 | disability:ti,ab OR disabled:ti,ab | 268,385 |
| 13 | 'activity of daily living':ti,ab | 3,263 |
| 14 | 'activities of daily living':ti,ab | 40,031 |
| 15 | 'activity of daily life':ti,ab | 341 |
| 16 | 'activities of daily life':ti,ab | 1,857 |
| 17 | 'daily life activity'/exp/mj | 14,822 |
| 18 | participation:ti,ab | 207,496 |
| 19 | activity:ti,ab | 3,491,683 |
| 20 | limitation:ti,ab | 120,101 |
| 21 | #19 AND #20 | 16,085 |
| 22 | #11 OR #12 OR #13 OR #14 OR #15 OR #16 OR #17 OR #18 OR #21 | 2,131,790 |
| 23 | prevalence:ti,ab | 964,018 |
| 24 | frequency:ti,ab | 1,138,741 |
| 25 | burden:ti,ab | 352,672 |
| 26 | 'randomized controlled trial'/exp/mj | 11,937 |
| 27 | 'randomization'/exp | 91,820 |
| 28 | inciden*:ti,ab | 1,362,309 |
| 29 | incidence:ti,ab | 1,157,883 |
| 30 | risk:ti,ab | 3,334,654 |
| 31 | 'hazard ratio':ti,ab | 154,212 |
| 32 | 'hazard ratios':ti,ab | 45,812 |
| 33 | odds:ti,ab | 460,665 |
| 34 | chance:ti,ab | 101,595 |
| 35 | percent:ti,ab | 402,184 |
| 36 | percentage:ti,ab | 577,947 |
| 37 | percentages:ti,ab | 92,283 |
| 38 | rate:ti,ab | 3,018,423 |
| 39 | rates:ti,ab | 1,571,532 |
| 40 | occurrence:ti,ab | 499,939 |
| 41 | responsiveness:ti,ab | 131,335 |
| 42 | 'sensitivity to change':ti,ab | 3,234 |
| 43 | frequencies:ti,ab | 237,267 |
| 44 | #23 OR #24 OR #25 OR #26 OR #27 OR #28 OR #29 OR #30 OR #31 OR #32 OR #33 OR #34 OR #35 OR #36 OR #37 OR #38 OR #39 OR #40OR #41 OR #42 OR #43 | 10,403,809 |
| 45 | older:ti,ab | 653,439 |
| 46 | elderly:ti,ab | 367,177 |
| 47 | geriatric:ti,ab | 69,535 |
| 48 | 'aged'/exp | 3,321,306 |
| 49 | aged:ti,ab | 854,083 |
| 50 | frail:ti,ab | 21,314 |
| 51 | frails:ti,ab | 28 |
| 52 | frailty:ti,ab | 25,936 |
| 53 | 'frailty'/exp | 15,719 |
| 54 | frailness:ti,ab | 29 |
| 55 | ageing:ti,ab | 60,209 |
| 56 | senior:ti,ab | 51,231 |
| 57 | elders:ti,ab | 11,925 |
| 58 | #45 OR #46 OR #47 OR #48 OR #49 OR #50 OR #51 OR #52 OR #53 OR #54 OR #55 OR #56 OR #57 | 5,394,076 |
| 59 | 'hospital associated':ti,ab | 1,830 |
| 60 | 'hospital-associated':ti,ab | 1,836 |
| 61 | 'hospital acquired':ti,ab | 15,633 |
| 62 | 'hospital-acquired':ti,ab | 15,725 |
| 63 | 'hospital related':ti,ab | 1,235 |
| 64 | 'hospital-related':ti,ab | 1,241 |
| 65 | 'hospitalization associated':ti,ab | 442 |
| 66 | 'hospitalization-associated':ti,ab | 444 |
| 67 | 'hospitalisation-associated':ti,ab | 58 |
| 68 | 'hospitalisation associated':ti,ab | 58 |
| 69 | 'hospitalization acquired':ti,ab | 4 |
| 70 | 'hospitalization-acquired':ti,ab | 4 |
| 71 | 'hospitalisation acquired':ti,ab | 0 |
| 72 | 'hospitalisation-acquired':ti,ab | 0 |
| 73 | 'hospitalization related':ti,ab | 394 |
| 74 | 'hospitalization-related':ti,ab | 396 |
| 75 | 'hospitalisation related':ti,ab | 45 |
| 76 | 'hospitalisation-related':ti,ab | 45 |
| 77 | 'before hospitalisation':ti,ab | 202 |
| 78 | 'before hospitalization':ti,ab | 1,692 |
| 79 | 'after hospitalisation':ti,ab | 671 |
| 80 | 'after hospitalization':ti,ab | 6,053 |
| 81 | functional:ti,ab AND ((deterioration NEAR/3 hospital*):ti,ab) | 83 |
| 82 | ((hospital* NEAR/3 functional):ti,ab) AND loss:ti,ab | 241 |
| 83 | functional:ti,ab AND ((status NEAR/3 hospital*):ti,ab) | 859 |
| 84 | (los* NEAR/3 independence):ti,ab | 2,106 |
| 85 | (exposure NEAR/2 hospital*):ti,ab | 1,197 |
| 86 | functional:ti,ab AND ((decline NEAR/4 hospital*):ti,ab) | 582 |
| 87 | #59 OR #60 OR #61 OR #62 OR #63 OR #64 OR #65 OR #66 OR #67 OR #68 OR #69 OR #70 OR #71 OR #72 OR #73 OR #74 OR #75 OR #76OR #77 OR #78 OR #79 OR #80 OR #81 OR #82 OR #83 OR #84 OR #85 OR #86 | 21,314 |
| 88 | #10 AND #22 AND #44 AND #58 AND #87 | 1,557 |

Cochrane search.

| **ID** | **Search** | **Results the 26 Aug 2021** |
| --- | --- | --- |
| 1 | hospitalisation:ti,ab | 30,002 |
| 2 | MeSH descriptor: [Hospitalization] explode all trees | 14,616 |
| 3 | hospitalization:ti,ab | 29,933 |
| 4 | hospital:ti,ab | 129,129 |
| 5 | hospitalized:ti,ab | 16,701 |
| 6 | hospitalised:ti,ab | 16,701 |
| 7 | in-hospital:ti,ab | 12,291 |
| 8 | inhospital:ti,ab | 5,816 |
| 9 | inpatient:ti,ab | 12,747 |
| 10 | #1 OR #2 OR #3 OR #4 OR #5 OR #6 OR #7 OR #8 OR #9 | 167,548 |
| 11 | functional:ti,ab | 76,938 |
| 12 | disability:ti,ab | 31,499 |
| 13 | disabled:ti,ab | 1,712 |
| 14 | "activity of daily living":ti,ab | 711 |
| 15 | "activities of daily living":ti,ab | 7,311 |
| 16 | "activity of daily life":ti,ab | 88 |
| 17 | "activities of daily life":ti,ab | 338 |
| 18 | MeSH descriptor: [Activities of Daily Living] explode all trees | 9,739 |
| 19 | participation:ti,ab | 30,692 |
| 20 | activity:ti,ab | 132,423 |
| 21 | limitation:ti,ab | 7,562 |
| 22 | #20 AND #21 | 1,315 |
| 23 | #11 OR #12 OR #13 OR #14 OR #15 OR #16 OR #17 OR #18 OR #19 OR #22 | 139,849 |
| 24 | prevalence:ti,ab | 33,961 |
| 25 | frequency:ti,ab | 79,109 |
| 26 | burden:ti,ab | 22,207 |
| 27 | MeSH descriptor: [Random Allocation] explode all trees | 20,643 |
| 28 | MeSH descriptor: [Randomized Controlled Trial] explode all trees | 119 |
| 29 | inciden*:ti,ab | 125,517 |
| 30 | incidence:ti,ab | 116,076 |
| 31 | risk:ti,ab | 219,370 |
| 32 | "hazard ratio":ti,ab | 22,039 |
| 33 | "hazard ratios":ti,ab | 3,476 |
| 34 | odds:ti,ab | 27,403 |
| 35 | chance:ti,ab | 8,943 |
| 36 | percent:ti,ab | 34,597 |
| 37 | percentage:ti,ab | 50,616 |
| 38 | percentages:ti,ab | 6,400 |
| 39 | rate:ti,ab | 282,297 |
| 40 | rates:ti,ab | 136,486 |
| 41 | occurrence:ti,ab | 27,342 |
| 42 | responsiveness:ti,ab | 7,597 |
| 43 | "sensitivity to change":ti,ab | 357 |
| 44 | frequencies:ti,ab | 7,361 |
| 45 | #24 OR #25 OR #26 OR #27 OR #28 OR #29 OR #30 OR #31 OR #32 OR #33 OR #34 OR #35 OR #36 OR #37 OR #38 OR #39 OR #40 OR #41 OR #42 OR #43 OR #44 | 728,891 |
| 46 | older:ti,ab | 59,544 |
| 47 | elderly:ti,ab | 45,936 |
| 48 | geriatric:ti,ab | 5,730 |
| 49 | MeSH descriptor: [Aged] explode all trees | 213,642 |
| 50 | aged:ti,ab | 118,657 |
| 51 | frail:ti,ab | 2,397 |
| 52 | frails:ti,ab | 4 |
| 53 | frailty:ti,ab | 2,066 |
| 54 | MeSH descriptor: [Frailty] explode all trees | 198 |
| 55 | frailness:ti,ab | 0 |
| 56 | ageing:ti,ab | 9,339 |
| 57 | senior:ti,ab | 3,235 |
| 58 | elders:ti,ab | 1,199 |
| 59 | #46 OR #47 OR #48 OR #49 OR #50 OR #51 OR #52 OR #53 OR #54 OR #55 OR #56 OR #57 OR #58 | 381,978 |
| 60 | "hospital associated":ti,ab | 99 |
| 61 | "hospital-associated":ti,ab | 99 |
| 62 | "hospital acquired":ti,ab | 823 |
| 63 | "hospital-acquired":ti,ab | 823 |
| 64 | "hospital related":ti,ab | 60 |
| 65 | "hospital-related":ti,ab | 60 |
| 66 | "hospitalization associated":ti,ab | 82 |
| 67 | "hospitalization-associated":ti,ab | 82 |
| 68 | "hospitalisation-associated":ti,ab | 82 |
| 69 | "hospitalisation associated":ti,ab | 82 |
| 70 | "hospitalization acquired":ti,ab | 1 |
| 71 | "hospitalization-acquired":ti,ab | 1 |
| 72 | "hospitalisation acquired":ti,ab | 1 |
| 73 | "hospitalisation-acquired":ti,ab | 1 |
| 74 | "hospitalization related":ti,ab | 58 |
| 75 | "hospitalization-related":ti,ab | 58 |
| 76 | "hospitalisation related":ti,ab | 58 |
| 77 | "hospitalisation-related":ti,ab | 58 |
| 78 | "before hospitalisation":ti,ab | 128 |
| 79 | "before hospitalization":ti,ab | 128 |
| 80 | "after hospitalisation":ti,ab | 785 |
| 81 | "after hospitalization":ti,ab | 785 |
| 82 | functional deterioration near/3 hospital*:ti,ab | 16 |
| 83 | hospital* near/3 functional loss:ti,ab | 93 |
| 84 | functional status near/3 hospital*:ti,ab | 134 |
| 85 | los* near/3 independence:ti,ab | 250 |
| 86 | exposure near/2 hospital*:ti,ab | 74 |
| 87 | functional decline near/4 hospital*:ti,ab | 62 |
| 88 | #60 OR #61 OR #62 OR #63 OR #64 OR #65 OR #66 OR #67 OR #68 OR #69 OR #70 OR #71 OR #72 OR #73 OR #74 OR #75 OR #76 OR #77 OR #78 OR #79 OR #80 OR #81 OR #82 OR #83 OR #84 OR #85 OR #86 OR #87 | 2,574 |
| 89 | #10 AND #23 AND #45 AND #59 AND #88 | 222 |

Additional material: Differences from protocol

The research question originally defined in the protocol, relating to the evaluation of which functional task is the most sensitive to detect changes in disability in hospitalized older adults, was withdrawn due to the lack of evidence in the literature on the method to be applied to assess this point.

It was decided not to integrate the grading system of the GRADE Working Group as predefined the evaluation of the body of evidence due to the lack of a guideline for incidence studies.

Additional material Table 2. Calculated and estimated incidences of HAD categorized per ADL task and set of tasks.

| **Study** | **Item** | **Response options** | **Criteria for response options** | **Baseline assessment (who, how & when)** | **Baseline prevalence of dependency** ^a^ | **Discharge assessment (who, how & when)** | **Discharge prevalence of dependency** | **Operationalization of HAD (item level)** | **Incidence HAD** |
| --- | --- | --- | --- | --- | --- | --- | --- | --- | --- |
| Covinsky 2000 (Covinsky et al., 2000) | Bathing (Katz et al., 1963) | I, D | I: ADL w/o help from person | BL<-2wk<- ADM; R: patient or proxy | PR: PS: 43%, AS: 0% | R: patient or proxy by phone 3 months after DC | PR: PS: 52% AS: 32% | Change from I to D | PR: 32% |
|  |  |  |  |  | PX: PS: 79%, AS: 0% |  | PX: PS: 90% AS: 70% |  | PX: 70% |
| Covinsky 2003 (Covinsky et al., 2003) | Bathing (Katz et al., 1963) | I, D | I: ADL w/o help from person | BL<-2wk<-ADM; R: patient or proxy | PS: 0%, AS: 0%, | R: patient or proxy at DC | PS: 24%, AS: 24% | Change from I to D | 24% ^c^ |
| Dharmarajan 2020 (Dharmarajan et al., 2020) | Bathing (Gill, 2014) | I, D | I: ADL w/o help from person | R: patient at last home-based assessment before hospitalization | PS: 14%, AS: 0% | R: patient or proxy by phone 1 month after DC | PS: 39%, AS: 25% ^b^ | Change from I to D | 25% ^b, c^ |
| Inouye 1993 (Inouye et al., 1993) | Bathing (Katz et al., 1963) | I, PA, TA | Partial assistance (PA): 1 body part assisted; D: > 1 body part | BL<-2wk<-ADM; R: patient | PS: 0%, AS: 0% | O: nurse at DC | PS: 10%, AS: 10% ^b^ | + 1 response option (I to PA or TA, or PA to TA) | 10% ^b, c^ |
| Mudge 2010 (Mudge et al., 2010) | Bathing (Katz et al., 1970) | I, D | I: ADL w/o help from person | BL<-2wk<-ADM; R: patient or proxy | PS: 21%, AS: 0% | O: Nurse at DC | PS: 47%, AS: 26% ^b^ | Change BL-DC prevalence | 26% ^b, c^ |
| Park 2021 (Park et al., 2021) | Bathing (Rockwood and Mitnitski, 2011) | I, D | I: ADL w/o help from person | BL<-30days<-ADM; R: patient or proxy | RB: PS: 0%, AS: 0% | R: patient or proxy by phone 1 month after BL | RB: PS: 16%, AS: 16% ^b^ | Change BL-DC prevalence | RB: 16% ^b, c^ |
|  |  |  |  |  | PF: PS: 3%, AS: 0% |  | PF: PS: 31%, AS: 28% ^b^ |  | PF: 28% ^b, c^ |
|  |  |  |  |  | MMF: PS: 62%, AS: 0% |  | MMF: PS: 90%, AS: 28% ^b^ |  | MMF: 28% ^b, c^ |
|  |  |  |  |  | SF: PS: 100%, AS: 0% |  | SF: PS: 100%, AS: 0% ^b^ |  | SF: 0% ^b, c^ |
| Sager 1996 (Sager et al., 1996) | Bathing (Katz et al., 1970) | I, D | I: ADL w/o help from person | BL<-2wk<-ADM; R: patient or proxy | PS: 27%, AS: 0% | R: patient or proxy at DC | PS: 81%, AS: 54% ^b^ | Change BL-DC prevalence | 54% ^b, c^ |
| Zelada 2009 (Zelada et al., 2009) | Bathing (Katz et al., 1963) | I, D | I: ADL w/o help from person | BL<-2wk<-ADM; R: patient or proxy | Geriatric unit: PS: NR, AS: 0% | R: patient or proxy at DC | Geriatric unit:  PS: NR, AS: 12% ^d^ | Change from I to D | Geriatric unit: 12% ^d^ |
|  |  |  |  |  | Usual unit: PS: NR, AS: 0% |  | Usual unit: PS: NR, AS: 21% ^d^ |  | Usual unit: 21% ^d^ |
| Covinsky 2000 (Covinsky et al., 2000) | Dressing (Katz et al., 1963) | I, D | I: ADL w/o help from person | BL<-2wk<-ADM; R: patient or proxy | PR: PS: 32%, AS: 0% | R: patient or proxy by phone 3 months after DC | PR: PS: 40%, AS: 27% | Change from I to D | PR: 27% |
|  |  |  |  |  | PX: PS: 70%, AS: 0% |  | PX: PS: 86%, AS: 66% |  | PX: 66% |
| Covinsky 2003 (Covinsky et al., 2003) | Dressing (Katz et al., 1963) | I, D | I: ADL w/o help from person | BL<-2wk<-ADM; R: patient or proxy | PS: 0%, AS: 0% | R: patient or proxy at DC | PS: 24%, AS: 24% | Change from I to D | 24% ^c^ |
| Dharmarajan 2020 (Dharmarajan et al., 2020) | Dressing (Gill, 2014) | I, D | I: ADL w/o help from person | R: patient at last home-based assessment before hospitalization | PS: 7%, AS: 0% | R: patient or proxy by phone 1 month after DC | PS: 25%, AS: 18% ^b^ | Change from I to D | 18% ^b, c^ |
| Inouye 1993 (Inouye et al., 1993) | Dressing (Katz et al., 1963) | I, PA, TA | PA: assisted tying shoes; D: assisted to dress/undress | BL<-2wk<-ADM; R: patient | PS: 0%, AS: 0% | O: nurse at DC | PS: 11%, AS: 11% ^b^ | + 1 response option (I to PA or TA, or PA to TA) | 11% ^b, c^ |
| Mudge 2010 (Mudge et al., 2010) | Dressing (Katz et al., 1970) | I, D | I: ADL w/o help from person | BL<-2wk<-ADM; R: patient or proxy | PS: 20%, AS: 0% | O: Nurse at DC | PS: 46 %, AS: 26% ^b^ | Change BL-DC prevalence | 26% ^b, c^ |
| Park 2021 (Park et al., 2021) | Dressing (Rockwood and Mitnitski, 2011) | I, D | I: ADL w/o help from person | BL<-30days<-ADM; R: patient or proxy | RB: PS: 0%, AS: 0% | R: patient or proxy by phone 1 month after BL | RB: PS: 7%, AS: 7% ^b^ | Change BL-DC prevalence | RB: 7% ^b, c^ |
|  |  |  |  |  | PF: PS: 0%, AS: 0% |  | PF: PS: 19%, AS: 19% ^b^ |  | PF: 19% ^b, c^ |
|  |  |  |  |  | MMF: PS: 8%, AS: 0% |  | MMF: PS: 45%, AS: 37% ^b^ |  | MMF: 37% ^b, c^ |
|  |  |  |  |  | SF: PS: 86%, AS: 0% |  | SF: PS: 100%, AS: 14% ^b^ |  | SF: 14% ^b, c^ |
| Sager 1996 (Sager et al., 1996) | Dressing (Katz et al., 1970) | I, D | I: ADL w/o help from person | BL<-2wk<-ADM; R: patient or proxy | PS: 16%, AS: 0% | R: patient or proxy at DC | PS: 64%, AS: 48% ^b^ | Change BL-DC prevalence | 48% ^b, c^ |
| Zelada 2009 (Zelada et al., 2009) | Dressing (Katz et al., 1963) | I, D | I: ADL w/o help from person | BL<-2wk<-ADM; R: patient or proxy | Geriatric unit:  PS: NR, AS: 0% | R: patient or proxy at DC | Geriatric unit:  PS: NR, AS: 5% ^d^ | Change from I to D | Geriatric unit: 5% ^d^ |
|  |  |  |  |  | Usual unit:  PS: NR, AS: 0% |  | Usual unit:  PS: NR, AS: 23% ^d^ |  | Usual unit: 23% ^d^ |
| Covinsky 2000 (Covinsky et al., 2000) | Toileting (Katz et al., 1963) | I, D | I: ADL w/o help from person | BL<-2wk<-ADM; R: patient or proxy | PR: PS: 36%, AS: 0% | R: patient or proxy by phone 3 months after DC | PR: PS: 39%, AS: 27% | Change from I to D | PR: 27% |
|  |  |  |  |  | PX: PS: 71%, AS: 0% |  | PX: PS: 86%, AS: 70% |  | PX: 70% |
| Covinsky 2003 (Covinsky et al., 2003) | Toileting (Katz et al., 1963) | I, D | I: ADL w/o help from person | BL<-2wk<-ADM; R: patient or proxy | PS: 0%, AS: 0% | R: patient or proxy at DC | PS: 16%, AS: 16% | Change from I to D | 16% ^c^ |
| Inouye 1993 (Inouye et al., 1993) | Toileting (Katz et al., 1963) | I, PA, TA | PA: assisted going to toilet or cleaning or arranging clothes or use of night bedpan/commode; D: doesn’t go to toilet | BL<-2wk<-ADM; R: patient | PS: 0%, AS: 0% | O: nurse at DC | PS: 12%, AS: 12% ^b^ | + 1 response option (I to PA or TA, or PA to TA) | 12% ^b, c^ |
| Hirsch 1990 (Hirsch et al., 1990) | Toileting (Hirsch et al., 1990) | I, PA, TA | PA: slight help or bedpan sometimes; D: lots of help or bedpan all the time | BL<-2wk<-ADM; R: patient or proxy | PS: 22%, AS: 0% | R: patient or proxy at DC | PS: 82%, AS: 60% ^b^ | + 1 response option (I to PA or TA, or PA to TA) | 60% ^b, c^ |
| Mudge 2010 (Mudge et al., 2010) | Toileting (Katz et al., 1970) | I, D | I: ADL w/o help from person | BL<-2wk<-ADM; R: patient or proxy | PS: 10%, AS: 0% | O: Nurse at DC | PS: 24%, AS: 14% ^b^ | Change BL-DC prevalence | 14% ^b, c^ |
| Park 2021 (Park et al., 2021) | Toileting (Rockwood and Mitnitski, 2011) | I, D | I: ADL w/o help from person | BL<-30days<-ADM; R: patient or proxy | RB: PS: 0%, AS: 0% | R: patient or proxy by phone 1 month after BL | RB: PS: 9%, AS: 9% ^b^ | Change BL-DC prevalence | RB: 9% ^b, c^ |
|  |  |  |  |  | PF: PS: 0%, AS: 0% |  | PF: PS: 23%, AS: 23% ^b^ |  | PF: 23% ^b, c^ |
|  |  |  |  |  | MMF: PS: 24%, AS: 0% |  | MMF: PS: 58%, AS: 34% ^b^ |  | MMF: 34% ^b, c^ |
|  |  |  |  |  | SF: PS: 92%, AS: 0% |  | SF: PS: 100%, AS: 8% ^b^ |  | SF: 8% ^b, c^ |
| Sager 1996 (Sager et al., 1996) | Toileting (Katz et al., 1970) | I, D | I: ADL w/o help from person | BL<-2wk<-ADM; R: patient or proxy | PS: 8%, AS: 0% | R: patient or proxy at DC | PS: 44%, AS: 36% ^b^ | Change BL-DC prevalence | 36% ^b, c^ |
| Zelada 2009 (Zelada et al., 2009) | Toileting (Katz et al., 1963) | I, D | I: ADL w/o help from person | BL<-2wk<-ADM; R: patient or proxy | Geriatric unit:  PS: NR, AS: 0% | R: patient or proxy at DC | Geriatric unit:  PS: NR, AS: 4% ^d^ | Change from I to D | Geriatric unit: 4% ^d^ |
|  |  |  |  |  | Usual unit:  PS: NR, AS: 0% |  | Usual unit:  PS: NR, AS: 19% ^d^ |  | Usual unit: 19% ^d^ |
| Covinsky 2000 (Covinsky et al., 2000) | Eating (Katz et al., 1963) | I, D | I: ADL w/o help from person | BL<-2wk<-ADM; R: patient or proxy | PR: PS: 31%, AS: 0% | R: patient or proxy by phone 3 months after DC | PR: PS: 39%, AS: 30% | Change from I to D | PR: 30 % |
|  |  |  |  |  | PX: PS: 69%, AS: 0% |  | PX: PS: 80%, AS: 61% |  | PX: 61% |
| Covinsky 2003 (Covinsky et al., 2003) | Eating (Katz et al., 1963) | I, D | I: ADL w/o help from person | BL<-2wk<-ADM; R: patient or proxy | PS: 0%, AS: 0% | R: patient or proxy at DC | PS: 15%, AS: 15% | Change from I to D | 15% ^c^ |
| Inouye 1993 (Inouye et al., 1993) | Eating (Katz et al., 1963) | I, PA, TA | PA: assisted cutting meat or buttering bread; D: assisted feeding or fed partially/completely tube or intravenous fluids | BL<-2wk<-ADM; R: patient | PS: 0%, AS: 0% | O: nurse at DC | PS: 5%, AS: 5% ^b^ | + 1 response option (I to PA or TA, or PA to TA) | 5% ^b, c^ |
| Hirsch 1990 (Hirsch et al., 1990) | Eating (Hirsch et al., 1990) | I, PA, TA | PA: assisted cutting meat, buttering bread, opening milk carton, etc; D: untidy in feeding | BL<-2wk<-ADM; R: patient or proxy | PS: 30%, AS: 0% | R: patient or proxy at DC | PS: 66%, AS: 36% ^b^ | + 1 response option (I to PA or TA, or PA to TA) | 36% ^b, c^ |
| Mudge 2010 (Mudge et al., 2010) | Eating (Katz et al., 1970) | I, D | I: ADL w/o help from person | BL<-2wk<-ADM; R: patient or proxy | PS: 2%, AS: 0% | O: Nurse at DC | PS: 5%, AS: 3% ^b^ | Change BL-DC prevalence | 3% ^b, c^ |
| Park 2021 (Park et al., 2021) | Eating (Rockwood and Mitnitski, 2011) | I, D | I: ADL w/o help from person | BL<-30days<-ADM; R: patient or proxy | RB: PS: 0%, AS: 0% | R: patient or proxy by phone 1 month after BL | RB: PS: 7%, AS: 7% ^b^ | Change BL-DC prevalence | RB: 7% ^b, c^ |
|  |  |  |  |  | PF: PS: 0%, AS: 0% |  | PF: PS: 3%, AS: 3% ^b^ |  | PF: 3% ^b, c^ |
|  |  |  |  |  | MMF: PS: 0%, AS: 0% |  | MMF: PS: 10%, AS: 10% ^b^ |  | MMF: 10% ^b, c^ |
|  |  |  |  |  | SF: PS: 57%, AS: 0% |  | SF: PS: 75%, AS: 18% ^b^ |  | SF: 18% ^b, c^ |
| Sager 1996 (Sager et al., 1996) | Eating (Katz et al., 1970) | I, D | I: ADL w/o help from person | BL<-2wk<-ADM; R: patient or proxy | PS: 6%, AS: 0% | R: patient or proxy at DC | PS: 28%, AS: 22% ^b^ | Change BL-DC prevalence | 22% ^b, c^ |
| Zelada 2009 (Zelada et al., 2009) | Eating (Katz et al., 1963) | I, D | I: ADL w/o help from person | BL<-2wk<-ADM; R: patient or proxy | Geriatric unit:  PS: NR, AS: 0% | R: patient or proxy at DC | Geriatric unit:  PS: NR, AS: 0% ^d^ | Change from I to D | Geriatric unit: 0% ^d^ |
|  |  |  |  |  | Usual unit:  PS: NR, AS: 0% |  | Usual unit:  PS: NR, AS: 1% ^d^ |  | Usual unit: 1% ^d^ |
| Inouye 1993 (Inouye et al., 1993) | Grooming (Katz et al., 1970)^e^ | I, PA, TA | PA: assisted cutting meat or buttering bread; D: assisted feeding or fed partially/completely tube or intravenous fluids | BL<-2wk<-ADM; R: patient | PS: 0%, AS: 0% | O: nurse at DC | PS: 11%, AS: 11% ^b^ | + one response option (I to PA or TA, or PA to TA) | 11% ^b, c^ |
| Hirsch 1990 (Hirsch et al., 1990) | Grooming (Hirsch et al., 1990) | I, PA, TA | PA: needs minor help or supervision; D: needs total grooming | BL<-2wk<-ADM; R: patient or proxy | PS: 31%, AS: 0% | R: patient or proxy at DC | PS: 78%, AS: 47% ^b^ | + 1 response option (I to PA or TA, or PA to TA) | 47% ^b, c^ |
| Park 2021 (Park et al., 2021) | Grooming, (Rockwood and Mitnitski, 2011) | I, D | I: ADL w/o help from person | BL<-30days<-ADM; R: patient or proxy | RB: PS: 0%, AS: 0% | R: patient or proxy by phone 1 month after BL | RB: PS: 5%, AS: 5% ^b^ | Change BL-DC prevalence | RB: 5% ^b, c^ |
|  |  |  |  |  | PF: PS: 0%, AS: 0% |  | PF: PS: 14%, AS: 14% ^b^ |  | PF: 14% ^b, c^ |
|  |  |  |  |  | MMF: PS: 11%, AS: 0% |  | MMF: PS: 29%, AS: 18% ^b^ |  | MMF: 18% ^b, c^ |
|  |  |  |  |  | SF: PS: 79%, AS: 0% |  | SF: PS: 89%, AS: 10% ^b^ |  | SF: 10% ^b, c^ |
| Covinsky 2000 (Covinsky et al., 2000) | Transferring (Katz et al., 1963) | I, D | I: ADL w/o help from person | BL<-2wk<-ADM; R: patient or proxy | PR: PS: 30%, AS: 0% | R: patient or proxy by phone 3 months after DC | PR: PS: 36%, AS: 27% | Change from I to D | PR: 27% |
|  |  |  |  |  | PX: PS: 60%, AS: 0% |  | PX: PS: 77%, AS: 59% |  | PX: 59% |
| Covinsky 2003 (Covinsky et al., 2003) | Transferring (Katz et al., 1963) | I, D | I: ADL w/o help from person | BL<-2wk<-ADM; R: patient or proxy | PS: 0%, AS: 0% | R: patient or proxy at DC | PS: 24%, AS: 24% | Change from I to D | 24% ^c^ |
| Dharmarajan 2020 (Dharmarajan et al., 2020) | Transferring from a chair (Gill, 2014) | I, D | I: ADL w/o help from person | R: patient at last home-based assessment before hospitalization | PS: 5%, AS: 0% | R: patient or proxy by phone 1 month after DC | PS: 19%, AS: 14% ^b^ | Change from I to D | 14% ^b, c^ |
| Hirsch 1990 (Hirsch et al., 1990) | Transferring (Hirsch et al., 1990) | I, PA, TA | PA: moves in and out of bed or chair with minor physical assistance; D: bedbound unless receives major physical help | BL<-2wk<-ADM; R: patient or proxy | PS: 27%, AS: 0% | R: patient or proxy at DC | PS: 83%, AS: 56% ^b^ | + 1 response option (I to PA or TA, or PA to TA) | 56% ^b, c^ |
| Mudge 2010 (Mudge et al., 2010) | Transferring (Katz et al., 1970) | I, D | I: ADL w/o help from person | BL<-2wk<-ADM; R: patient or proxy | PS: 12%, AS: 0% | O: Nurse at DC | PS: 26%, AS: 14% ^b^ | Change BL-DC prevalence | 14% ^b, c^ |
| Park 2021 (Park et al., 2021) | Transferring (Rockwood and Mitnitski, 2011) | I, D | I: ADL w/o help from person | BL<-30days<-ADM; R: patient or proxy | RB: PS: 0%, AS: 0% | R: patient or proxy by phone 1 month after BL | RB: PS: 2%, AS: 2% ^b^ | Change BL-DC prevalence | RB: 2% ^b, c^ |
|  |  |  |  |  | PF: PS: 0%, AS: 0% |  | PF: PS: 8%, AS: 8% ^b^ |  | PF: 8% ^b, c^ |
|  |  |  |  |  | MMF: PS: 3%, AS: 0% |  | MMF: PS: 20%, AS: 17% ^b^ |  | MMF: 17% ^b, c^ |
|  |  |  |  |  | SF: PS: 62%, AS: 0% |  | SF: PS: 85%, AS: 23% ^b^ |  | SF: 23% ^b, c^ |
| Sager 1996 (Sager et al., 1996) | Transferring (Katz et al., 1970) | I, D | I: ADL w/o help from person | BL<-2wk<-ADM; R: patient or proxy | PS: 7%, AS: 0% | R: patient or proxy at DC | PS: 54%, AS: 47% ^b^ | Change BL-DC prevalence | 47% ^b, c^ |
| Zelada 2009 (Zelada et al., 2009) | Transferring (Katz et al., 1963) | I, D | I: ADL w/o help from person | B<-2wk<-ADM; R: patient or proxy | Geriatric unit:  PS: NR, AS: 0% | R: patient or proxy at DC | Geriatric unit:  PS: NR, AS: 3% ^d^ | Change from I to D | Geriatric unit: 3% ^d^ |
|  |  |  |  |  | Usual unit:  PS: NR, AS: 0% |  | Usual unit:  PS: NR, AS: 14% ^d^ |  | Usual unit: 14% ^d^ |
| Hirsch 1990 (Hirsch et al., 1990) | Incontinence of bowel or bladder (Hirsch et al., 1990) | I, PA, TA | PA: occasionally incontinent (1-4 times in last 48 hours); D: Frequently incontinent (>4 times in last 48 hours, has indwelling/condom catheter/requires intermittent catheter | BL<-2wk<-ADM; R: patient or proxy | PS: 48%, AS: 0% | R: patient or proxy at DC | PS: 52%, AS: 4% ^b^ | + 1 response option (I to PA or TA, or PA to TA) | 4% ^b, c^ |
| Zelada 2009 (Zelada et al., 2009) | Continence (Katz et al., 1963) | I, D | I: ADL w/o help from person | BL<-2wk<-ADM; R: patient or proxy | Geriatric unit:  PS: NR, AS: 0% | R: patient or proxy at DC | Geriatric unit:  PS: NR, AS: 2% ^d^ | Change from I to D | Geriatric unit: 2% ^d^ |
|  |  |  |  |  | Usual unit:  PS: NR, AS: 0% |  | Usual unit:  PS: NR, AS: 6% ^d^ |  | Usual unit: 6% ^d^ |
| Dharmarajan 2020 (Dharmarajan et al., 2020) | Walking (Gill, 2014) | I, D | I: ADL w/o help from person | R: patient at last home-based assessment before hospitalization | PS: 1%, AS: 0% | R: patient or proxy by phone 1 month after DC | PS: 19%, AS: 18% ^b^ | Change from I to D | 18% ^b, c^ |
| Hirsch 1990 (Hirsch et al., 1990) | Mobility (Hirsch et al., 1990) | I, PA, TA | PA: unsteady-needs supervision or minimal support; D: needs major help to walk or must be pushed in W/C | BL<-2wk<-ADM; R: patient or proxy | PS: 30%, AS: 0% | R: patient or proxy at DC | PS: 88%, AS: 58% ^b^ | + 1 response option (I to PA or TA, or PA to TA) | 58% ^b, c^ |
| Mudge 2010 (Mudge et al., 2010) | Mobility,  (Katz et al., 1970)^e^ | I, D | I: ADL w/o help from person | BL<-2wk<-ADM; R: patient or proxy | PS: 18%, AS: 0% | O: nurse at DC | PS: 37%, AS: 19% ^b^ | Change BL-DC prevalence | 19% ^b, c^ |
| Park 2021 (Park et al., 2021) | Walking (Rockwood and Mitnitski, 2011) | I, D | I: ADL w/o help from person | BL<-30days<-ADM; R: patient or proxy | RB: PS: 0%, AS: 0% | R: patient or proxy by phone 1 month after BL | RB: PS: 9%, AS: 9% ^b^ | Change BL-DC prevalence | RB: 9% ^b, c^ |
|  |  |  |  |  | PF: PS: 3%, AS: 0% |  | PF: PS: 25%, AS: 22% ^b^ |  | PF: 22% ^b, c^ |
|  |  |  |  |  | MMF: PS: 14%, AS: 0% |  | MMF: PS: 52%, AS: 38% ^b^ |  | MMF: 38% ^b, c^ |
|  |  |  |  |  | SF: PS: 91%, AS: 0% |  | SF: PS: 97%, AS: 6% ^b^ |  | SF: 6% ^b, c^ |
| Sager 1996 (Sager et al., 1996) | Walking across a room (Katz et al., 1970)^e^ | I, D | I: ADL w/o help from person | BL<-2wk<-ADM; R: patient or proxy | PS: 8%, AS: 0% | R: patient or proxy at DC | PS: 53%, AS: 45% ^b^ | Change BL-DC prevalence | 45% ^b, c^ |
| Park 2021 (Park et al., 2021) | Walking 1 km from (Nagi, 1976; Rosow and Breslau, 1966) included in the Frailty Index (Rockwood and Mitnitski, 2011) | I, D | I: ADL w/o help from person | BL<-30days<-ADM; R: patient or proxy | RB: PS: 16%, AS: 0% | R: patient or proxy by phone 1 month after BL | RB: PS: 40%, AS: 24% ^b^ | Change BL-DC prevalence | RB: 24% ^b, c^ |
|  |  |  |  |  | PF: PS: 64%, AS: 0% |  | PF: PS: 77%, AS: 13% ^b^ |  | PF: 13% ^b, c^ |
|  |  |  |  |  | MMF: PS: 88%, AS: 0% |  | MMF: PS: 96%, AS: 8% ^b^ |  | MMF: 8% ^b, c^ |
|  |  |  |  |  | SF: PS: 100%, AS: 0% |  | SF: PS: 100%, AS: 0% ^b^ |  | SF: 0% ^b, c^ |
| Park 2021 (Park et al., 2021) | Walking up and down a flight of stairs from (Nagi, 1976; Rosow and Breslau, 1966) included in the Frailty Index (Rockwood and Mitnitski, 2011) | I, D | I: ADL w/o help from person | BL<-30days<-ADM; R: patient or proxy | RB: PS: 4%, AS: 0% | R: patient or proxy by phone 1 month after BL | RB: PS: 25%, AS: 21% ^b^ | Change BL-DC prevalence | RB: 21% ^b, c^ |
|  |  |  |  |  | PF: PS: 38%, AS: 0% |  | PF: PS: 66%, AS: 27% ^b^ |  | PF: 27% ^b, c^ |
|  |  |  |  |  | MMF: PS: 71%, AS: 0% |  | MMF: PS: 84%, AS: 13% ^b^ |  | MMF: 13% ^b, c^ |
|  |  |  |  |  | SF: PS: 100%, AS: 0% |  | SF: PS: 100%, AS: 0% ^b^ |  | SF: 0% ^b, c^ |
| Mudge 2010 (Mudge et al., 2010) | Overall (Katz et al., 1970) | I, D | I: ADL w/o help from person | BL<-2wk<-ADM; R: patient or proxy | PS: 28%, AS: 28% NaR: 0% | O: nurse at DC | PS: 51%, AS: 51% | Change BL-DC prevalence | 40% |
| Sager 1996 (Sager et al., 1996) | Overall (Katz et al., 1970) | I, D | I: ADL w/o help from person | BL<-2wk<-ADM; R: patient or proxy | PS: 27%, AS: 27%, NaR: 0% | R: patient or proxy at DC | PS: NR, AS: NR | Change BL-DC prevalence | 43% |
| Inouye 1993 (Inouye et al., 1993) | Overall (Katz et al., 1963) | I, PA, TA | PA: assisted cutting meat or buttering bread; D: assisted feeding or fed partially/completely tube or intravenous fluids | BL<-2wk<-ADM; R: patient | PS: 35%, AS: 35%, NaR: 0% | O: nurse at DC | PS: NR, AS: NR | + 1 response option (I to PA or TA, or PA to TA) | 27% |
| Zelada 2009 (Zelada et al., 2009) | Overall (Katz et al., 1963) | I, D | I: ADL w/o help from person | BL<-2wk<-ADM; R: patient or proxy | Geriatric unit:  PS: 57%, AS: 57%, NaR: 0% | R: patient or proxy at DC | Geriatric unit:  PS: NR, AS: NR | Change from I to D | Geriatric unit: 19% ^d^ |
|  |  |  |  |  | Usual unit:  PS: 49%, AS:49%, NaR: 0% |  | Usual unit:  PS: NR, AS: NR |  | Usual unit: 40% ^d^ |
| Covinsky 2003 (Covinsky et al., 2003) | Overall (Katz et al., 1963) | I, D | I: ADL w/o help from person | BL<-2wk<-ADM; R: patient or proxy | PS: 33%, AS: 33%, NaR: 0% | R: patient or proxy at DC | PS: 35%, AS: 35% | Change from I to D | 35% |

ADM, admission; AS, analyzed sample; BL<-2wk<-ADM, retrospective assessment at hospital admission on ADL ability 2 weeks before admission; BL, baseline; D, dependent; DC, discharge; HAD, hospital-associated disability; I, independent; MMF, mild-to-moderate frailty; NaR, not at risk in the analyzed sample; NR, not reported; O, observed; PA, partial assistance/or using materials (e.g. bedpan); PF, pre-frail; PR, patient reported; PX, proxy reported; R, reported; RB, robust; PS, published sample; SF, severe frailty; TA, total assistance (i.e. dependent) ; wk, weeks; w/o, without. a, two prevalences are presented: the published sample reported by the authors and the analysis sample in our analysis;

b, this is the difference between discharge prevalence and baseline prevalence. However, this does not correspond to HAD because some of the patients who were dependent at baseline might have improved. Hence, this estimate of HAD is a mix of two groups, (1) the increase in HAD in the group that was independent at baseline, and (2) the improvement in the group of patients who were dependent at baseline; c, Newly dependent was not available; d, newly dependent are available, but the incidence was calculated with a denominator that included not only *N* at risk, but also people who could not develop further dependency; e, The authors provide the reference for the Katz Index, but this item does not appear in the original version of the instrument.
