# Supplementary figures and images for: Assessment tools and incidence of hospital-associated disability in older adults: a rapid systematic review
Source: PeerJ. 2023 Oct 19;11:e16036. doi: 10.7717/peerj.16036 (PMC10590575; doi:10.7717/peerj.16036)

# JBI – Checklist study ratings

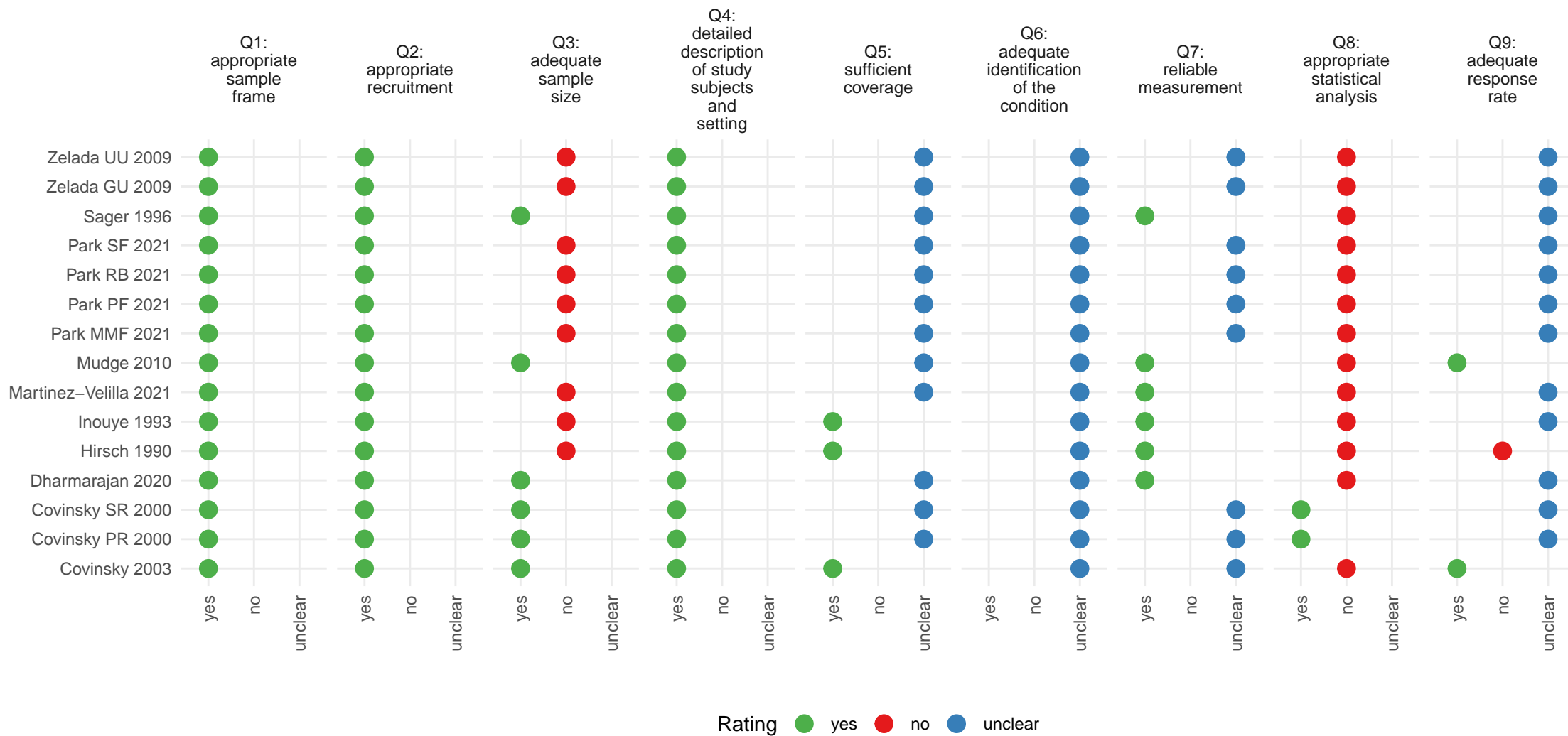

Supplement: Supplemental Information 2 [file peerj-11-16036-s002.pdf]
