# Supplementary material for: Assessment tools and incidence of hospital-associated disability in older adults: a rapid systematic review
Source: PeerJ. 2023 Oct 19;11:e16036. doi: 10.7717/peerj.16036 (PMC10590575; doi:10.7717/peerj.16036)
Supplement: Supplemental Information 4 [file peerj-11-16036-s004.docx]

**The rationale for conducting the systematic review / meta-analysis**

During hospitalization, older adults (over 65 years of age) are at risk of developing functional decline unrelated to the condition for which they were admitted. The loss of independence in at least one activity of daily living is referred to as hospital-associated disability (HAD) (Covinsky et al. 2011). HAD is important because it is triggered by multiple factors that increase the risk of its occurrence. The risk factors described in the literature are dehydration, poor nutrition (Nagae et al. 2023), immobilization (Brown et al. 2004), restricted mobility due to medical devices (Chen et al. 2022), reduced mobility (Lindenberger et al. 2003), polypharmacy (Steinman & Hanlon 2010), allostatic overload (Duan-Porter et al. 2019), maladaptive cognitive perception (Walker et al. 1987), decreased reserve and morbidity (Covinsky et al. 2011). The number of older people will double by 2025 compared to 2015 (WHO 2022), so it is likely that the number of people developing HAD will increase as well. The problem is not negligible, the consequences of decline can be dramatic and lead to increased use of health resources, nursing home placement and death (Covinsky et al. 2003; Sager et al. 1996).

There is currently no consensus on which tool should be used to assess functional decline in these patients, which activity of daily living (ADL) tasks should be included, how the assessment should be performed (self-reported or performance-based), and what time-frame should be considered (Buurman et al. 2011). Monitoring disability in activities of daily living is important to measure as it is predictive of admission to a nursing home, the need for home adaptation, hospitalization and the use of home help (Edemekong et al., 2022).

Several studies assessed ADL with different tools and provide sufficient information to calculate the incidence of hospital-associated disability. It is important to know the extent of HAD so that measures can be put in place to prevent and limit this problem. New data are emerging, and new publications have recently appeared on ADLs in the older population, justifying the need for a systematic review (Dharmarajan et al. 2020; Park et al. 2021).

**The contribution that it makes to knowledge in light of previously published related reports, including other meta-analyses and systematic reviews**

Previous studies have highlighted methodological issues in assessing functional decline (Buurman et al. 2011; Loyd et al. 2020). However, there is currently no consensus on which tool should be used to assess functional decline in hospitalized older patients over 65 years , which ADL tasks should be included, how the assessment should be performed (self-reported or performance-based), and what time-frame should be considered (Buurman et al. 2011). In this review we investigated the tools used to assess activities of daily living in older hospitalized patients in acute care by conducting a rapid systematic review.

Despite significant changes in the healthcare system over the years (reduction in length of stay and emphasis on quality) (Brown 2020), functional decline due to hospitalization remains a significant problem. A previous study highlighted the magnitude of the HAD problem, and reported that the overall prevalence of HAD among older adults admitted to an acute care hospital is 30% (Loyd et al. 2020).

However, to our knowledge, no other review has neither investigated the overall incidence of HAD nor the incidence at the item level in a population of older patients over 65 years hospitalized for acute care.

Several studies assessed ADL in older adults and provided sufficient information to calculate the overall HAD incidence. The pooled incidence of HAD on the total score was 37%. We believe that this value of HAD is overestimated due to a combination of disease-related disability and HAD, while measurement tools may also present some limitations.

The result of the incidence of HAD at the level of the individual task could not be analyzed by a meta-analysis. We were able to show that the incidence of the individual items varied significantly depending on whether it was patient-reported or proxy-reported. For example, 32% of patients said they were unable to wash themselves, while 70% of disabilities were reported by proxy. This review highlights the lack of systematic reporting of data used to calculate HAD incidence.

**References**

Brown CJ. 2020. After Three Decades of Study, Hospital-Associated Disability Remains a Common Problem. *J Am Geriatr Soc* 68:465-466. 10.1111/jgs.16349

Brown CJ, Friedkin RJ, and Inouye SK. 2004. Prevalence and outcomes of low mobility in hospitalized older patients. *J Am Geriatr Soc* 52:1263-1270. 10.1111/j.1532-5415.2004.52354.x

Buurman BM, van Munster BC, Korevaar JC, de Haan RJ, and de Rooij SE. 2011. Variability in measuring (instrumental) activities of daily living functioning and functional decline in hospitalized older medical patients: a systematic review. *Journal of clinical epidemiology* 64:619-627. 10.1016/j.jclinepi.2010.07.005

Chen Y, Almirall-Sanchez A, Mockler D, Adrion E, Dominguez-Vivero C, and Romero-Ortuno R. 2022. Hospital-associated deconditioning: Not only physical, but also cognitive. *Int J Geriatr Psychiatry* 37. 10.1002/gps.5687

Covinsky KE, Palmer RM, Fortinsky RH, Counsell SR, Stewart AL, Kresevic D, Burant CJ, and Landefeld CS. 2003. Loss of independence in activities of daily living in older adults hospitalized with medical illnesses: increased vulnerability with age. *Journal of the American Geriatrics Society* 51:451-458. 10.1046/j.1532-5415.2003.51152.x

Covinsky KE, Pierluissi E, and Johnston CB. 2011. Hospitalization-associated disability: "She was probably able to ambulate, but I'm not sure". *Jama* 306:1782-1793. 10.1001/jama.2011.1556

Dharmarajan K, Han L, Gahbauer EA, Leo-Summers LS, and Gill TM. 2020. Disability and Recovery After Hospitalization for Medical Illness Among Community-Living Older Persons: A Prospective Cohort Study. *J Am Geriatr Soc* 68:486-495. 10.1111/jgs.16350

Duan-Porter W, Vo TN, Ullman K, Langsetmo L, Strotmeyer ES, Taylor BC, Santanasto AJ, Cawthon PM, Newman AB, Simonsick EM, Waters TM, and Ensrud KE. 2019. Hospitalization-Associated Change in Gait Speed and Risk of Functional Limitations for Older Adults. *J Gerontol A Biol Sci Med Sci* 74:1657-1663. 10.1093/gerona/glz027

Lindenberger EC, Landefeld CS, Sands LP, Counsell SR, Fortinsky RH, Palmer RM, Kresevic DM, and Covinsky KE. 2003. Unsteadiness reported by older hospitalized patients predicts functional decline. *J Am Geriatr Soc* 51:621-626. 10.1034/j.1600-0579.2003.00205.x

Loyd C, Markland AD, Zhang Y, Fowler M, Harper S, Wright NC, Carter CS, Buford TW, Smith CH, Kennedy R, and Brown CJ. 2020. Prevalence of Hospital-Associated Disability in Older Adults: A Meta-analysis. *J Am Med Dir Assoc* 21:455-461 e455. 10.1016/j.jamda.2019.09.015

Nagae M, Umegaki H, Komiya H, Fujisawa C, Watanabe K, Yamada Y, and Miyahara S. 2023. Dehydration and hospital-associated disability in acute hospitalized older adults. *Eur Geriatr Med* 14:113-121. 10.1007/s41999-022-00722-5

Park CM, Kim W, Rhim HC, Lee ES, Kim JH, Cho KH, and Kim DH. 2021. Frailty and hospitalization-associated disability after pneumonia: A prospective cohort study. *BMC Geriatr* 21:111. 10.1186/s12877-021-02049-5

Sager MA, Franke T, Inouye SK, Landefeld CS, Morgan TM, Rudberg MA, Sebens H, and Winograd CH. 1996. Functional outcomes of acute medical illness and hospitalization in older persons. *Archives of internal medicine* 156:645-652.

Steinman MA, and Hanlon JT. 2010. Managing medications in clinically complex elders: "There's got to be a happy medium". *Jama* 304:1592-1601. 10.1001/jama.2010.1482

Walker FBt, Novack DH, Kaiser DL, Knight A, and Oblinger P. 1987. Anxiety and depression among medical and surgical patients nearing hospital discharge. *J Gen Intern Med* 2:99-101. 10.1007/BF02596305

WHO. 2022. Ageing and health. *Available at* [*https://www.who.int/news-room/fact-sheets/detail/ageing-and-health*](https://www.who.int/news-room/fact-sheets/detail/ageing-and-health).
